# Inhibitory immune checkpoints PDCD-1 and LAG-3 hypermethylation may reduce the risk of colorectal cancer

**DOI:** 10.1186/s10020-021-00373-5

**Published:** 2021-09-20

**Authors:** Yuanyuan Zhang, Lei zhang, Hongru Sun, Ying Liu, Jing Xu, Hao Huang, Jinming Fu, Ding Zhang, Tian Tian, Yashuang Zhao, Guiyu Wang

**Affiliations:** 1 Department of Epidemiology, Public Health College of Harbin Medical University, 157 Baojian Street, Nangang District, Harbin, 150081 Heilongjiang People’s Republic of China; 2 Department of Colorectal Cancer Surgery, The Second Affiliated Hospital of Harbin Medical University, 246 Xuefu Street, Nangang District, Harbin, 150001 Heilongjiang People’s Republic of China

**Keywords:** Colorectal cancer, Peripheral blood leukocyte, *PDCD-1*, *LAG-3*, DNA methylation

## Abstract

**Background:**

Changes in DNA methylation of immunosuppressive checkpoints may impact express and consequently affect antigen processing and presentation by tumor cells and facilitates evasion of immunosurveillance and lead to colorectal cancer (CRC). This study is to investigate the effect of *PDCD-1*, *LAG-3* methylation statuses in peripheral blood leukocytes on CRC risk.

**Methods:**

GSE51032 dataset from Gene Expression Omnibus comprised of 166 CRC patients and 424 normal samples was used to identify significantly differentially methylated CpG sites of the two genes. A case–control study with 390 CRC patients and 397 cancer-free controls was carried out to validate the relationship between the methylation levels of the two genes and CRC susceptibility and then estimated their interactions with environmental factors on CRC risk.

**Results:**

In the GSE51032 dataset, cg06291111 (*PDCD-1*) and cg10191002 (*LAG-3*) were screened as the candidate CpG sites for the following study. There were significant associations between hypermethylation of *PDCD-1* and *LAG-3* and lower risk of CRC (OR_adj_ = 0.322, 95% CI 0.197–0.528; OR_adj_ = 0.666, 95% CI 0.446–0.5996, respectively). Moreover, the results in case–control study showed similar trend, that hypermethylation of *PDCD-1* and *LAG-3* were associated with lower CRC risk (OR_adj_ = 0.448, 95% CI 0.322–0.622; OR_adj_ = 0.417, 95% CI 0.301–0.578, respectively). A synergistic interaction between *LAG-3* hypermethylation and intake of eggs on CRC risk was observed. There were combination effects between hypermethylation of *PDCD-1* and *LAG-3* and environmental factors on CRC risk.

**Conclusions:**

*PDCD-1* and *LAG-3* may potentially serve as blood-based predictive biomarkers for CRC risk.

**Supplementary Information:**

The online version contains supplementary material available at 10.1186/s10020-021-00373-5.

## Background

Worldwide, colorectal cancer (CRC) is the third most common cancer in terms of incidence, accounting for nearly 1.8 million new cases annually (Bray et al. 2018). By 2030, the global burden of CRC is expected to increase by 60%, and new cases will reach 2.2 million (Arnold et al. 2017).

The occurrence process of CRC results from internal and external factors interaction. At present, the association between immune evasion and the risk of CRC attracts great interest of researchers. Dual host-protective and tumor-promoting function of immune system are identified as cancer immunoediting, which is the process consisting of three sequential phases: immunosurveillance, equilibrium, and escape (Pernot et al. 2014; Dunn et al. 2004). Studies have revealed that the development of CRC is related to the epigenetic silencing of immunosuppressive checkpoints (ICs), which may impact antigen processing and presentation by tumor cells and facilitates evasion of immunosurveillance. Epigenetic regulation is the major mechanisms behind ICs expression (Héninger et al. 2015). Aberrant expression of ICs in cancer creates an immunosuppressive microenvironment, supporting the immune escape of tumor cells. Numerous negative regulatory mechanisms, which can inhibit anti-tumor responses are implemented through the expression of various ICs on immune and tumor cells (Elashi 2019).

As one of the most widely studied and recognized ICs, *PDCD-1*, involved in almost every aspect of immune responses (Okazaki et al. 2007), dampens the immune response of activated T cells and mediates the inhibition of T cells during long-term antigen exposure (Flemming 2012). Polymorphisms in *PDCD-1* have been reported to be associated with the risk of CRC (Zhang 2016). *LAG-3*, another IC that is expected to be targeted in the clinic, consequently garnering considerable interest and scrutiny. Serve as a surface protein, *LAG-3* is expressed by immune cells normally (Andrews et al. 2017; Marin-Acevedo et al. 2018), which play a vital role in evasion of the host immune response in kinds of tumors. *LAG-3* is required for optimal T cell regulation and homeostasis. Persistent antigen-stimulation in tumors including CRC (Camisaschi et al. 2010; Llosa et al. 2015), leads to anomalous expression of *LAG-3*, promoting T cell exhaustion (Ruffo 2019). V.S. Nair et al. discovered abnormal expression levels and anomalous methylation patterns of various ICs including *PDCD-1* and *LAG-3* in colorectal tumor tissues compared with normal tissues (Sasidharan Nair et al. 2018). Furthermore, co-expression between *PDCD-1* and *LAG-3* in environments containing either tumor, self-Ag, or chronic infection can result in T cells with poor effector function (Woo et al. 2012; Lucas et al. 2011; Matsuzaki et al. 2010; Grosso et al. 2009). Until now, the research in this field is mostly carried out at the tumor tissue level, whether methylation statuses of *PDCD-1* and *LAG-3* in PBL could be involved in the anomalous expression of ICs and related to CRC risk remain unclear.

We therefore carried out this study to discover significantly differentially methylated CpG sites (DMCs) of *PDCD-1* and *LAG-3* using the GSE51032 dataset from the European Prospective Investigation into Cancer and Nutrition (EPIC) study and then screened the DMCs of the two genes located in CpG island and had the least *P* value as the candidate CpG sites to further explore the relationship between the methylation of the two genes and CRC risk in the case–control study. Moreover, we investigated the combination effect between the methylation of the two genes on CRC risk. Since DNA methylation alterations in leukocytes may reflect epigenetic modifications (gene expression), environmental exposures, or interactions between these factors that increase cancer susceptibility (Kitkumthorn et al. 2012; Huang et al. 2012; Walters et al. 2013; Nan 2013; Ally et al. 2009; Kaaks et al. 2009; Miroglio et al. 2010; Gao et al. 2012; Luo et al. 2016). We then further explored combination and interaction effects between *PDCD-1, LAG-3* methylation statuses and environmental factors on CRC risk in PBL.

## Methods

### Data sources

**Fig. 1:**
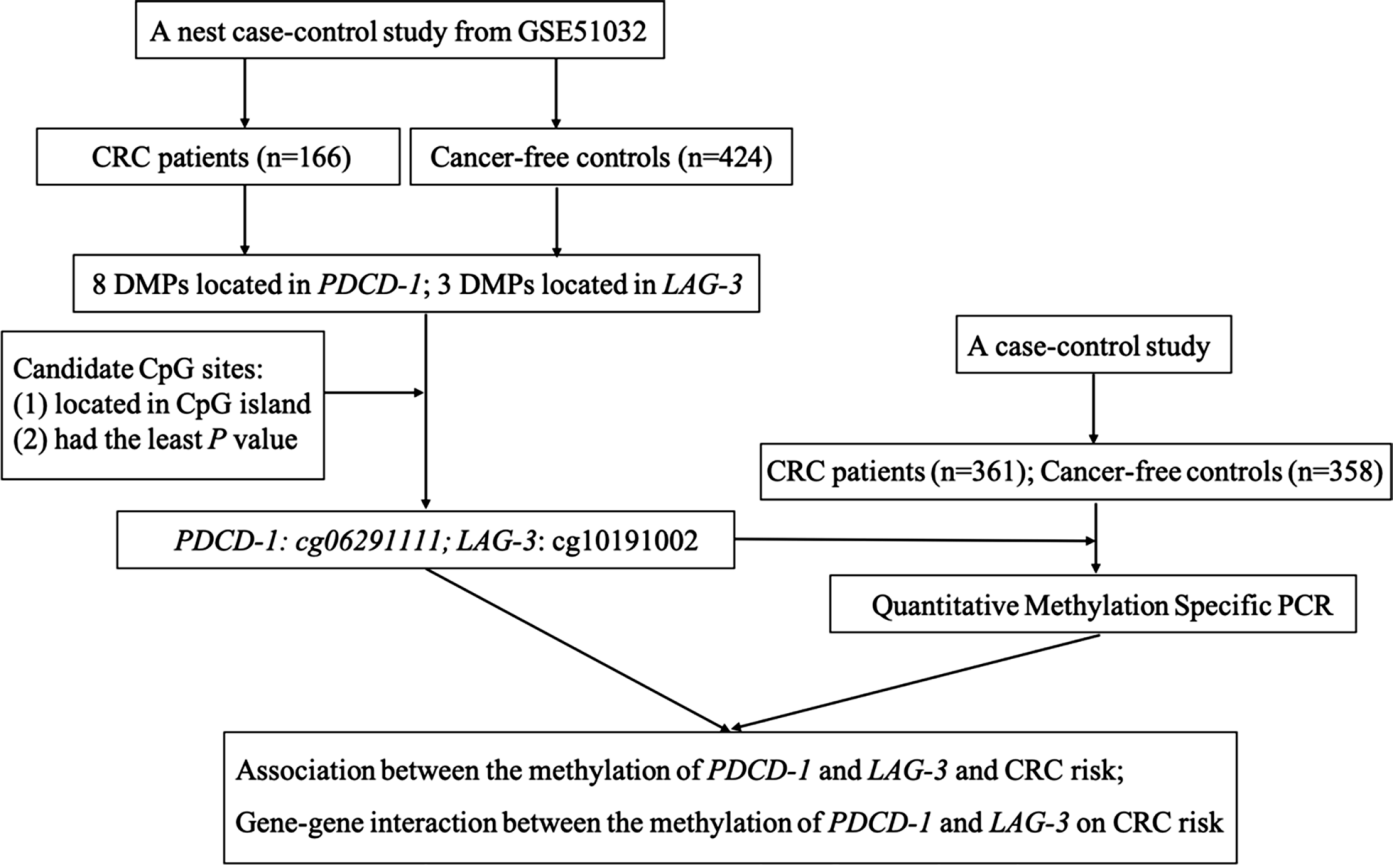
Flow chart of the study

The workflow of the study is summarized in Fig. 1. Firstly, GSE51032 dataset from the EPIC study, a nested case–control study, that was designed to investigate the relationships between genetic and environmental factors and the incidences of different cancers was used to screen the DMCs of *PDCD-1* and *LAG-3* as the candidate CpG sites for the further study. Secondly, a case–control study was carried out to explore the relationship between the methylation of the two genes and CRC risk. Thirdly, we further explored combination and interaction effects between *PDCD-1* and *LAG-3* methylation statuses and environmental factors on CRC risk.

DNA methylation data of the peripheral blood leukocytes for CRC were downloaded from the GEO database (https://www.ncbi.nlm.nih.gov/geo/) with the GEO accession number GSE51032 (166 CRC patients and 424 normal samples). The platform was Illumina Infinium HumanMethylation450 BeadChip. The methylation levels of each CpG site were represented by beta-value, which was the ratio between methylated probe intensities and total intensities.

### Data preprocessing and DMCs selection

Firstly, the CpG sites with missing values greater than 10% in the samples were removed. Secondly, probes were filtered based on the following conditions: (i) probes with < 3 beads in at least 5% of samples examined; (ii) probes with a detection *P* > 0.01; (iii) non-CpG probes; (iv) single-nucleotide polymorphism (SNP)-related probes; (v) multi-hit probes; (vi) probes located in X and Y chromosome. Thirdly, all missing data were imputed using the k-nearest neighbor imputation method. Beta-mixture quantile normalization (BMIQ) was applied for normalization. DMCs related to CRC were identified using minfi package in R with false discovery rate (FDR) less than 0.05.

### Study subjects and sample collection

We then carried out a hospital-based case–control study including 390 primary CRC patients who were diagnosed at the Second Affiliated Hospital of Harbin Medical University and the Third Affiliated Hospital of Harbin Medical University from 2015 to 2017, and 397 cancer-free control individuals enrolled from the Second Affiliated Hospital of Harbin Medical University during the same period. The patients with neuroendocrine carcinoma, malignant melanoma, non-Hodgkin’s lymphoma, gastrointestinal stromal tumors, or Lynch syndrome and the controls with a history of gastrointestinal disease based on self-reporting were excluded. Peripheral blood (5 ml) of every subject was collected before surgery or before treatment and immediately stored at −80 °C. Due to the lack of DNA, the methylation levels of *PDCD-1* were measured in only 389 cases and 386 controls.

### Genomic DNA extraction and sodium bisulfate modification

The blood samples were centrifuged at 1600*g* for 10 min to separate the plasma and the buffy coats. We extracted genomic DNA from the buffy coats using the QIAamp DNA Blood Mini kit (Qiagen, Hilden, Germany) and bisulfite-modified using the EpiTect Plus DNA Bisulfite Kit (Qiagen, Hilden, Germany) according to the manufacturer’s instructions. DNA quantity was measured using NanoDrop 2000 spectrophotometer (Thermo Fisher, USA).

### Data collection

Through in-person interviews, all subjects completed a structured questionnaire with information on demographic characteristics (i.e., age, gender, and education), lifestyles (i.e., alcohol drinking, cigarette smoking, and physical activity), dietary status within the 12 months before the interview (i.e., fruits and vegetables, fried food, overnight food) and family history of CRC. The questions were modified from the questionnaire of Shu et al. (Shu et al. 2004). This study was approved by the ethical committee of Harbin Medical University. We obtained written informed consent from all subjects and performed in accordance with the ethical standards in the 1964 Declaration of Helsinki and its later amendments.

### Quantitative methylation specific PCR (QMSP)

The detected CpG sites of the two genes were identified by the GEO dataset. The methylation statuses of *PDCD-1* and *LAG-3* were detected using quantitative methylation specific PCR (QMSP) on a LightCycler480 system (Roche Applied Science, Mannheim, Germany). We analyzed the methylation statuses of *PDCD-1* and *LAG-3* by Abs Quant/2nd Derivative Max software (version 2.0). Each PCR mixture consisted of a total volume of 10 μL containing 5 μL LightCycler480 High-Resolution Melting Master Mix (Roche Applied Science, Mannheim, Germany), 0.5 μL primer, approximately 10 ng bisulfite-modified template DNA, 3.4 μL of PCR-grade water. The PCR conditions were showed in Additional file 1: Table S1. We constructed a series of plasmid standards (5 × 10^7^, 5 × 10^6^, 5 × 10^5^, 5 × 10^4^, 5 × 10^3^, 5 × 10^2^, 5 × 10^1^ copies/μL) on a background of diluted positive standards (Bisulfite Converted Universal Methylated Human DNA Standards; Zymo Research, D5015) as standard curves. The standard curves were obtained by setting the cycle threshold (Ct) to the ordinate and the logarithm of the standard concentration to the abscissa. When the methylated standard concentrations were 5 × 10^7^, 5 × 10^6^, 5 × 10^5^, 5 × 10^4^, 5 × 10^3^, 5 × 10^2^, 5 × 10^1^ copies/μL, the Cts of *PDCD-1* were 13.51, 16.75, 20.05, 23.38, 28.05, 29.59 (The sample range of pre-tests were 5 × 107–5 × 102 copies/μL, Tm = 79 ± 0.5 ℃), respectively (Additional file 1: Fig S1); the Cts of *LAG-3* were 14.14, 17.62, 21.08, 24.67, 28.17, 31.18, 33.1 (Tm = 80 ± 0.5 ℃) respectively (Additional file 1: Fig S2); the Cts of ACTB were 13.83, 17.33, 20.71, 24.24, 27.72 (The sample range of pre-tests were 5 × 107–5 × 103 copies/μL, Tm = 76 ± 0.5 ℃), respectively (Additional file 1: Fig S3). The amplification efficiency was 0.9 < e < 1.0.

The methylation ratio (QMSP value) was defined as the ratio of the fluorescence emission intensity values for the target gene-specific PCR products to those of the *ACTB* (Reference gene) and then multiplied by 100 for easier tabulation. Additionally, two blank control (non-template control) samples were included in each batch, and all reactions were performed in duplicate. A third trial would be conducted if the two trials presented inconsistent results.

### Statistical analysis

To evaluate the homogeneity between cases and controls, two-sample t-tests for the continuous variables and chi-square (χ2) tests for the categorical variables were performed. We used the receiver operating characteristic (ROC) curve to determine the cut-off value to categorize all subjects into hypomethylation group and hypermethylation group. Methylation level in the target gene higher than the cut-off value was defined as hypermethylation; Otherwise, the level was defined as hypomethylation. Univariate and multivariate logistic regressions were applied to estimate the associations between environmental factors, genes methylation in PBL and their interaction on CRC risk with crude and adjusted estimate Odds Ratios (ORs) and 95% confidence intervals (CIs). We applied crossover analysis to evaluate the interaction effects of environmental factors and methylation of *PDCD-1* and *LAG-3*, and the interaction effect between methylation of *PDCD-1* and *LAG-3* on the risk of CRC. The statistical power for the study was calculated using PASS 15 (NCSS lnc., USA). Statistical analyses were performed using STATA 14 (Stata Corp., USA) and R version 3.5.1. *P* values < 0.05 were considered statistically significant.

## Results

### Identification of DMCs and the association between gene methylation and CRC risk in GEO dataset

The basic characteristics of CRC patients and controls in in GEO dataset were shown in Additional file 1: Table S2. We adjusted for age and gender in the following analyses. Eight DMCs located in *PDCD-1* (cg09031938, cg03903296, cg06291111, cg07281781, cg10057601, cg11532131, cg04789125, cg10994870) and four DMCs located in *LAG-3* (cg10191002, cg04153135, cg06157570, cg14292870) were associated with CRC risk in GEO dataset*.* Genomic information of 12 CpG sites were shown in Table 1. The CpG sites located in CpG island and had the least *P* value were screened as the candidate sites for the following study (*PDCD-1*: cg06291111; *LAG-3*: cg10191002). As shown in Table 2, hypermethylation of *PDCD-1* and *LAG-3* were associated with a lower risk of CRC (OR_adj_ = 0.322, 95% CI 0.197–0.528, *P* < 0.001; OR_adj_ = 0.666, 95% CI 0.446–0.5996, *P* = 0.048, respectively).

**Table 1:** Genomic information of 12 CpG sites for *PDCD-1* and *LAG-3* in GEO dataset

Gene Symbol	CpG	Chromosome	Relation to island	*P* value
*PDCD-1*	cg09031938	chr2	Island	2.661E−04
	cg03903296	chr2	Island	4.067E−07
	**cg06291111**	**chr2**	**Island**	**1.798E**−**07**
	cg07281781	chr2	Island	8.854E−05
	cg10057601	chr2	Shore	3.169E−02
	cg11532131	chr2	Shore	1.780E−02
	cg04789125	chr2	Shore	5.368E−03
	cg10994870	chr2	Island	5.111E−03
*LAG-3*	**cg10191002**	**chr12**	**Island**	**3.961E**−**02**
	cg04153135	chr12	Shore	2.355E−02
	cg06157570	chr12	Shelf	8.326E−03
	cg14292870	chr12	Opensea	2.019E−03

The final screened CpG sites were indicated in bold

**Table 2:** Associations between gene methylation and the CRC risk in GEO dataset

Gene	Hypomethylation	Hypermethylation	OR (95%CI)	*P value	OR_adj_ (95%CI)	*P value
*PDCD-1* (cg06291111)						
CRCs (n = 166), n%	142 (85.54%)	24 (14.46%)		**< 0.0001**		**< 0.0001**
Controls (n = 424), n%	267 (62.97%)	157 (37.03%)	0.287 (0.179–0.462)		0.322 (0.197–0.528)	
*LAG-3* (cg10191002)						
CRCs (n = 166), n%	66 (39.76%)	100 (60.24%)		**0.004**		**0.048**
Controls (n = 424), n%	116 (27.36%)	308 (72.64%)	0.571 (0.391–0.832)		0.666 (0.446–0.996)	

CRC, colorectal cancer; CI confidence interval; OR odds ratio; OR_adj_, adjusted for age, gender

^*^P values < 0.05 were considered statistically significant  and were indicated in bold

### Basic demographic characteristics of the cases and controls in the validation study

A total of 361 CRC patients and 358 controls were comprised in the study. The basic characteristics of all subjects were shown in Table 3. The distribution of age was significantly different between cases and controls, which was adjusted in following analyses. Related reports supported that estrogen delays the occurrence of CRC in females for 7–8 years (Lieberman et al. 2005). Although there is no statistical difference in gender distribution between the groups, we also adjusted for gender in the following analyses. Moreover, the distributions of environmental factors in all subjects were shown in Additional file 1: Table S3.

**Table 3:** Distribution of the basic characteristics of CRC patients and controls

Variables	CRCs (n = 361), n%	Controls (n = 358), n%	*P value
Age (years)			**< 0.0001**
Mean ± SD	60.38 ± 10.28	55.82 ± 11.92	**< 0.0001**
≤ 50	53 (14.68%)	104 (29.05%)	
50-	114 (31.58%)	102 (28.49%)	
60-	135 (37.40%)	116 (32.40%)	
> 70	59 (16.34%)	36 (10.06%)	
Gender			0.111
Male	225 (62.33%)	202 (56.42%)	
Female	136 (37.67%)	156 (43.58%)	
BMI			0.146
Mean ± SD	23.37 ± 4.36	23.81 ± 3.84	
< 18.5	20 (5.54%)	16 (4.47%)	
18.5-	107 (29.64%)	104 (29.05%)	
≥ 23	234 (64.82%)	238 (66.48%)	
Education			0.080
Primary school and below	161 (44.60%)	134 (37.43%)	
Junior senior and above	200 (55.40%)	224 (62.57%)	
Ethic group			0.847
Han	349 (96.68%)	344 (96.09%)	
Others	12 (3.32%)	14 (3.91%)	
Marriage status			0.896
Married	331 (90.94%)	325 (90.78%)	
Others	30 (9.06%)	33 (9.22%)	
Occupation			0.507
White collar	256 (70.91%)	262 (73.18%)	
Blue collar	105 (29.09%)	96 (26.82%)	
Family history of CRC			0.138
No	318 (88.09%)	328 (91.62%)	
Yes	43 (11.91%)	30 (8.38%)	
Alcohol drinking			**< 0.0001**
No	204 (56.51%)	252 (70.39%)	
Yes	157 (43.49%)	106 (29.61%)	
Smoking			0.668
No	253 (70.08%)	269 (75.14%)	
Yes	108 (29.92%)	89 (24.86%)	

*P values < 0.05 were considered statistically significant and were indicated in bold

CRC, colorectal cancer; SD, standard deviation; BMI, body mass index

### The association between gene methylation and CRC risk

The cut-off values of *PDCD-1* and *LAG-3* were 22.94% and 1.46%, respectively (Additional file 1: Fig S8). For *PDCD-1*, 59.13% cases (230/389) and 76.17% controls (294/386) were hypermethylated. Hypermethylation of *PDCD-1* was associated with a lower risk of CRC (OR_adj_ = 0.448, 95% CI 0.322–0.622, *P* < 0.001). For *LAG-3*, 56.67% cases (221/390) and 72.80% controls (289/397) were hypermethylated. Hypermethylation of *LAG-3* was also statistically associated with a lower risk of CRC (OR_adj_ = 0.417, 95% CI 0.301–0.578, *P* < 0.001) (Table 4, Additional file 1: Fig S4–S6).

**Table 4:** Association between methylation levels of *PDCD-1*, *LAG-3* in PBL and CRC risk

Gene	Hypomethylation	Hypermethylation	OR (95%CI)	*P value	OR_adj_ (95%CI)	*P value
*PDCD-1*						
CRCs (n = 389), n%	159 (40.87%)	230 (59.13%)		**< 0.0001**		**< 0.0001**
Controls (n = 386), n%	92 (23.83%)	294 (76.17%)	0.453 (0.332–0.617)		0.448 (0.322–0.622)	
*LAG-3*						
CRCs (n = 390), n%	169 (43.33%)	221 (56.67%)		**< 0.0001**		**< 0.0001**
Controls (n = 397), n%	108 (27.20%)	289 (72.80%)	0.489 (0.363–0.659)		0.417 (0.301–0.578)	

^*^P values < 0.05 were considered statistically significant and were indicated in bold

CRC, colorectal cancer; CI, confidence interval; OR, odds ratio; OR_adj_: adjusted for age, gender

In order to eliminate variants caused by differences repeat of tests, we conducted quality control and calculated the methylation level by taking the average of all coefficients and slopes. The results showed that the hypermethylation of *PDCD-1* and *LAG-3* were still associated with the lower risk of CRC with similar ORs (Additional file 1: Table S4).

### Age stratification analysis

There were significant associations between hypermethylation of *PDCD-1* and *LAG-3* and reduced CRC risk in young subjects (< 60 years) (*PDCD-1*: OR = 0.441, 95% CI 0.289–0.673, *P* < 0.001; *LAG-3*: OR = 0.336, 95% CI 0.215–0.525, *P* < 0.001), as well as in old subjects (≥ 60 years) (*PDCD-1*: OR = 0.457, 95% CI 0.278–0.754, *P* < 0.001; *LAG-3*: OR = 0.584, 95% CI 0.368–0.926, *P* = 0.022) (Table 5). There was no statistically significant difference between young and old subjects (*P* > 0.05 for all comparisons) in the associations between hypermethylation of these two genes and CRC risk. In GEO dataset, we observed the similar results (Additional file 1: Table S5). The associations between hypermethylation of *PDCD-1* and *LAG-3* and CRC risk stratified by different environmental factors were shown in Additional file 1: Table S6.

**Table 5:** Associations between the methylation of individual genes and CRC risk stratified by age

Subgroup	No. of hypermethylation (CRCs/controls)	No. of hypomethylation (CRCs/controls)	OR (95% CI)	*P* value^a^	*P* value^b^
*PDCD-1*					
Age, years					
< 60	102/166	78/56	0.441 (0.289–0.673)	**< 0.0001**	
≥ 60	110/105	71/31	0.457 (0.278–0.754)	**0.002**	0.914
*LAG-3*					
Age, years					
< 60	105/179	75/43	0.336 (0.215–0.525)	**< 0.0001**	
≥ 60	98/91	83/45	0.584 (0.368–0.926)	**0.022**	0.092

CRC, colorectal cancer; CI, confidence interval; OR, odds ratio

^a^*P* values were calculated using Logistic regression analysis, *P* values < 0.025 were considered statistically significant and  were indicated in bold

^b^Test for heterogeity between ORs was conducted by fixed effect models with STATA (version 14), *P* values < 0.05 were considered statistically significant

### Combination effect between the methylation of PDCD-1 and LAG-3 on CRC risk

A combination effect between *PDCD-1* and *LAG-3* methylation on CRC risk was observed (OR_eg_ = 0.217, 95% CI 0.127–0.370, *P* < 0.001). We didn’t observe significant interaction between methylation levels of *PDCD-1* and *LAG-3* on CRC risk (OR_i_ = 0.683, 95% CI 0.341–1.371, *P* = 0.286) (Table 6). In the GEO dataset, we observed the similar results (OR_eg_ = 0.135, 95% CI 0.067–0.271, *P* < 0.001; OR_i_ = 1.017, 95% CI 0.383–2.676, *P* = 0.980).

**Table 6:** Effects of combination and interaction between methylation of *PDCD-1* and *LAG-3*

Gene	*PDCD-1*		Interaction	
	Hypomethylation	Hypermethylation		
	OR_eg_ (95% CI)		OR_i_ (95% CI)	*P value
*LAG-3*				
Initial findings				
Hypomethylation		0.579 (0.326–1.028)		
Hypermethylation	0.548 (0.307–0.978)	0.217 (0.127–0.370)	0.683 (0.341–1.371)	0.283
GEO dataset				
Hypomethylation		0.264 (0.128–0.545)		
Hypermethylation	0.503 (0.324–0.782)	0.135 (0.067–0.271)	1.017 (0.383–2.676)	0.980

CI, confidence interval; OR, odds ratio

^*^P values < 0.05 were considered statistically significant

### Effects between environmental factors and PDCD-1 and LAG-3 methylation on CRC Risk

Significant combination effects between hypermethylation of *PDCD-1* and *LAG-3* and higher consumption of alcohol, overnight food, salty food, hot-scalded food, pork, beef, fowl, pickled cabbage and smoked and baked food on increased CRC risk were observed. Moreover, a significant combination effect between hypermethylation of *LAG-3* and higher intake of eggs on the reduction of CRC risk was observed (Additional file 1: Table S7–S8).

Synergistic interaction between *LAG-3* hypermethylation and higher intake of eggs on reducing CRC risk was observed (OR_i_ = 0.389, 95% CI 0.190–0.794, *P* = 0.010). We didn’t find the interaction between other environmental factors and *PDCD-1* methylation on CRC risk (Additional file 1: Table S7–S8).

## Discussion

In our study, we identified the DMCs of *PDCD-1* and *LAG-3* in the GSE51032 dataset and validated the relationship between the methylation levels of the two genes (*PDCD-1*: cg06291111; *LAG-3*: cg10191002) and CRC risk in a case–control study. We found that hypermethylation of *PDCD-1* and *LAG-3* in WBC-derived DNA were associated with reducing the risk of CRC. There were significant interaction and combination effects between hypermethylation of the two genes and environmental factors on CRC risk. We also observed a combination effect between *PDCD-1* and *LAG-3* methylation on CRC risk.

Epigenetic alterations of ICs such as aberrant methylation/demethylation pattern may alter gene expression and tumorigenesis (Elashi 2019). In order to check DNA epigenetic modifications behind the abnormal expression of ICs, V.S. Nair et.al. checked the expression of demethylation enzymes (TETs) and methylation enzymes (DNMTs) in the tumor tissue and normal tissue and found that the expression of demethylation enzymes was significantly higher and methylation enzymes were lower in tumor tissue. Evidence showed that the TET protein level was upregulated in solid tumors (Ficz et al. 2014). These data prompted us to check the CpG methylation profile of the promoter regions of ICs. Moreover, Leukocyte DNA methylation statuses of some genes may be associated with the susceptibility or risk of CRC (Gao et al. 2012). Therefore, the methylation levels of ICs in PBL may reflect the individual's susceptibility to CRC. Interestingly studies claimed that a strong synergy between the *PDCD-1* and *LAG-3* inhibitory pathways in tolerance to both self and tumor antigens and argued strongly that dual blockade of these molecules represents a promising combinatorial strategy for cancer, suggesting that they may contribute to T cell apoptosis and reduce autoimmune function jointly, induce tumor-mediated immune suppression (Woo et al. 2012; Lucas et al. 2011; Matsuzaki et al. 2010; Grosso et al. 2009). In our study, we measured the methylation levels and interaction effect of *PDCD-1* and *LAG-3* in nearly 800 PBL samples, including 397 CRCs and 390 controls. We observed that the protective effects of hypermethylation of *PDCD-1* and *LAG-3* in PBL, and a statistically significant combination effect between the hypermethylation of *PDCD-1* and *LAG-3*, which was similar to the results in the GEO dataset. Notably, the combination effect of *PDCD-1* and *LAG-3* in the leukocytes of CRC and the epigenetic modifications are important to understand the complex inhibitory immune mechanisms involved in the risk of CRC.

The Results of the GEO dataset, TCGA database (Additional file 1: Fig S7) and previous studies (Elashi 2019; Sasidharan Nair et al. 2018) showed that: compared with normal tissue and PBL samples from healthy controls, methylation status of *PDCD-1* in tumor tissues and PBL samples from CRC patients were in concordance with transcriptomic expression in CRC: the more the hypomethylation, the higher the expression, but *LAG-3* were not completely consistent in tissue and PBL samples. Thus, blood-derived DNA methylation measurements may not always represent the levels of colorectal tissue methylation (Li et al. 2013; McKay et al. 2011). Although all somatic cells in a given individual are genetically identical, different cell types form highly different anatomical structures and perform widely different physiological functions (Christensen 2009). It is speculated that in the process of tissue differentiation and development, the transcription relevant control regions in the genome are selectively demethylated or hypermethylated. So that a set of restricted genes can be transcribed within a given tissue (Walsh et al. 1999). Compared with other tissues, the methylation pattern in DNA obtained from blood may be more "plastic", because blood is very close to environmental effects (such as lifestyle) (McKay et al. 2011).

Some changes in methylation closely correlate with age which may provide markers for biological aging and play an important part in cancer. The genome continues to undergo programmed variation in methylation after birth in response to environmental inputs, serving as a memory that could affect aging and predisposition to cancer (Christensen 2009; Teschendorff et al. 2010; Jones et al. 2015). Because age is a key factor in cancer risk and gene methylation, we speculated that age might be a confounding factor, which might impact CRC risk. Remarkably, we did not find any statistically significant difference between the young subjects group (< 60 years) and the old subjects group (≥ 60 years) (*P* > 0.05 for all comparisons) for the associations between methylation of these two genes and CRC risk. It may imply that the methylations of these two genes are independent biomarkers for the susceptible of CRC.

Altered DNA methylation is associated with environmental exposures encountered throughout life (Walsh et al. 1999). In our study, we observed that *LAG-3* hypermethylation and higher intake of eggs (≥ 3 numbers/week) could synergistically reduce the risk of CRC. Vitamin B2 of eggs has antioxidant properties that can serve as a cofactor to enhance one-carbon metabolism, maintain intestinal mucosal stability, and has been implicated in lowering CRC risk (Yoon et al. 2016). Moreover, eggs are also rich in a variety of trace elements, such as zinc (Stepien et al. 2017), which have a role in reducing the risk of cancer. Besides, egg yolk can enrich conjugated linoleic acid, which has a wide range of biological functions, such as cancer inhibition (Bhattacharya et al. 2006; Larsson et al. 2005) and immune enhancement (Bassaganya-Riera et al. 2012). Therefore, DNA methylation changes in white blood cells may reflect epigenetic modification, human immune system, environmental exposure or the interaction among these factors on the risk of CRC.

However, there are still some limitations in our study. Firstly, we did not distinguish the type of cells in leukocytes. Different types of leukocytes may have different status of DNA methylation in tumor carcinogenesis. Nevertheless, studies focused on the methylation status of DNA from different leukocyte subtypes have suggested that confounding by leukocyte subtypes is potentially a minor issue that would not affect the DNA methylation level in the peripheral blood (Zilbauer et al. 2013). Secondly, our research results are based on a case–control study and cannot provide confirmation that whether abnormal changes of methylation statuses of *PDCD-1* and *LAG-3* are the preparatory epigenetic event of CRC or cancer-derived consequences; However, the GSE51032 dataset is from a nested case–control study of the prospective EPIC-Italy cohort (Cordero et al. 2015), in which the blood samples were collected 74.1 months (range from 0.2 to 172.8 months) prior to CRC diagnosis. This dataset can directly confirm the temporal relationship between methylation changes and tumorigenesis. Our results are consistent with the GEO dataset. The third limitation of the present study is the fact that the information about subjects that were recalled. The information of exposed environment factors may not reflect the real frequencies and amount. Nevertheless, the interviewers tried their best to collect the exact information. Finally, the sample size in the stratified analysis is relatively small, which may limit the statistic power in our research. Normally, the statistic power reaching to 0.8 is often considered an acceptable threshold. The statistical power for the age stratification analysis in our study was 0.82, which was close to 0.8. Therefore, more studies involving larger samples may be needed to improve the statistical power.

## Conclusion

The methylation levels of *PDCD-1* and *LAG-3* might be the blood-based predictive biomarkers for identifying individuals at lower risk of developing CRC. Moreover, gene-environment interaction may play a vital role in CRC risk.

## Supplementary Information

**Additional file 1: Fig. S1. ** Quantitative Methylation Specific PCR (QMSP) for the detection of *PDCD-1* methylation in standard samples. (A) Amplification curves of *PDCD-1* gene methylation standards. (B) Melting curves of serial dilutions of methylated DNA from the *PDCD-1* gene. (C) Standard curve used in the QMSP assay of *PDCD-1* gene. **Fig S2.** Quantitative Methylation Specific PCR (QMSP) for the detection of *LAG-3* methylation in standard samples. (A) Amplification curves of *LAG-3* gene methylation standards. (B) Melting curves of serial dilutions of methylated DNA from the *LAG-3* gene. (C) Standard curve used in the QMSP assay of *LAG-3* gene. **Fig. S3.** Quantitative Methylation Specific PCR (QMSP) for the detection of ACTB methylation in standard samples. (A) Amplification curves of *ACTB* gene methylation standards. (B) Melting curves of serial dilutions of methylated DNA from the *ACTB* gene. (C) Standard curve used in the QMSP assay of *ACTB* gene. **Fig S4.** Quantitative Methylation Specific PCR (QMSP) for the detection of *PDCD-1* methylation in PBL samples. (A) Amplification curves used in the PDCD-1 QMSP assay. (B) Melting curves of PBL samples from the *PDCD-1* gene. **Fig S5.** Quantitative Methylation Specific PCR (QMSP) for the detection of *LAG-3* methylation in PBL samples. (A) Amplification curves used in the *LAG-3* QMSP assay. (B) Melting curves of PBL samples from the *LAG-3* gene. **Fig S6.** Quantitative Methylation Specific PCR (QMSP) for the detection of *ACTB* methylation in PBL samples. (A) Amplification curves used in the ACTB QMSP assay. (B) Melting curves of PBL samples from the *ACTB* gene. **Fig S7.** Gene expression and DNA methylation levels of PDCD-1, *LAG-3* in TCGA database. (A) Expression of PDCD-1 gene. (B) Expression of *LAG-3* gene. (C) Methylation level of PDCD-1 gene. (D) Methylation level of *LAG-3* gene. **Fig S8.** ROC curves of *PDCD-1*, *LAG-3* in the case-control study. (A) cg06291111. (B) cg10191002 (C) PDCD-1 gene (D) *LAG-3* gene. **Table S1.** Primer sequence, amplicon size and reaction conditions. **Table S2.** Distribution of the basic characteristics of CRC patients and controls in in GEO dataset. **Table S3.** Distribution of the environmental factors of the CRC patients and controls. **Table S4.** Association between CRC risk and *PDCD-1*, *LAG-3* methylation levels in PBL calculated by using the average of coefficients and slopes of all standard curves. **Table S5.** Association between the methylation levels of *PDCD-1*, *LAG-3* and CRC risk stratified by age in GEO dataset. **Table S6.** Association between the methylation levels of *PDCD-1*, *LAG-3* and CRC risk stratified by different environmental factors in case-control study. **Table S7.**. Effects of combination and interaction between environmental factors and methylation of *PDCD-1* on the risk of CRC. **Table S8.** Effects of combination and interaction between environmental factors and methylation of *LAG-3* on the risk of CRC.

## Data Availability

The data generated or analyzed during the present study are available from the corresponding author on reasonable request.

## Abbreviations

CRC: Colorectal Cancer
*PDCD-1*: Programmed Cell Death-1
*LAG-3*: Lymphocyte Activation Gene-3
PBL: Peripheral Blood Leukocytes
ICs: Immunosuppressive Checkpoints
GEO: Gene Expression Omnibus
DMCs: Methylated Cpg Sites
EPIC: European Prospective Investigation into Cancer and Nutrition
SNP: Single-Nucleotide Polymorphism
BMIQ: Beta-Mixture Quantile Normalization
FDR: False Discovery Rate
QMSP: Quantitative Methylation Specific PCR
ROC: Receiver Operating Characteristic
ORs: Odds Ratios
Cis: Confidence Intervals
TETs: Demethylation Enzymes
DNMTs: Methylation Enzymes
WBC: White Blood Cell
CEA: Carcinoembryonic Antigen
CA19-9: Carbohydrate Antigen 19-9
