# Supplementary material for: Inhibitory immune checkpoints PDCD-1 and LAG-3 hypermethylation may reduce the risk of colorectal cancer
Source: Mol Med. 2021 Sep 20;27:114. doi: 10.1186/s10020-021-00373-5 (PMC8454079; doi:10.1186/s10020-021-00373-5)
Supplement: Supplementary file 1 — Additional file 1: Fig. S1. Quantitative Methylation Specific PCR (QMSP) for the detection of PDCD-1 methylation in standard samples. (A) Amplification curves of PDCD-1 gene methylation standards. (B) Melting curves of serial dilutions of methylated DNA from the PDCD-1 gene. (C) Standard curve used in the QMSP assay of PDCD-1 gene. Fig S2. Quantitative Methylation Specific PCR (QMSP) for the detection of LAG-3 methylation in standard samples. (A) Amplification curves of LAG-3 gene methylation standards. (B) Melting curves of serial dilutions of methylated DNA from the LAG-3 gene. (C) Standard curve used in the QMSP assay of LAG-3 gene. Fig. S3. Quantitative Methylation Specific PCR (QMSP) for the detection of ACTB methylation in standard samples. (A) Amplification curves of ACTB gene methylation standards. (B) Melting curves of serial dilutions of methylated DNA from the ACTB gene. (C) Standard curve used in the QMSP assay of ACTB gene. Fig S4. Quantitative Methylation Specific PCR (QMSP) for the detection of PDCD-1 methylation in PBL samples. (A) Amplification curves used in the PDCD-1 QMSP assay. (B) Melting curves of PBL samples from the PDCD-1 gene. Fig S5. Quantitative Methylation Specific PCR (QMSP) for the detection of LAG-3 methylation in PBL samples. (A) Amplification curves used in the LAG-3 QMSP assay. (B) Melting curves of PBL samples from the LAG-3 gene. Fig S6. Quantitative Methylation Specific PCR (QMSP) for the detection of ACTB methylation in PBL samples. (A) Amplification curves used in the ACTB QMSP assay. (B) Melting curves of PBL samples from the ACTB gene. Fig S7. Gene expression and DNA methylation levels of PDCD-1, LAG-3 in TCGA database. (A) Expression of PDCD-1 gene. (B) Expression of LAG-3 gene. (C) Methylation level of PDCD-1 gene. (D) Methylation level of LAG-3 gene. Fig S8. ROC curves of PDCD-1, LAG-3 in the case-control study. (A) cg06291111. (B) cg10191002 (C) PDCD-1 gene (D) LAG-3 gene. Table S1. Primer sequence, amplicon size and re [file 10020_2021_373_MOESM1_ESM.docx]

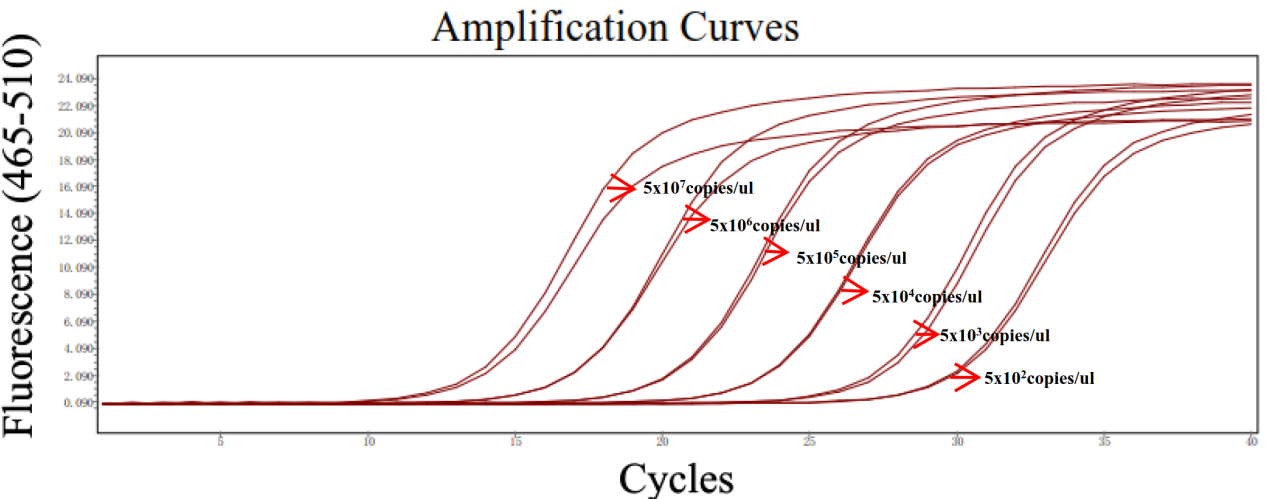


**B**

**A**


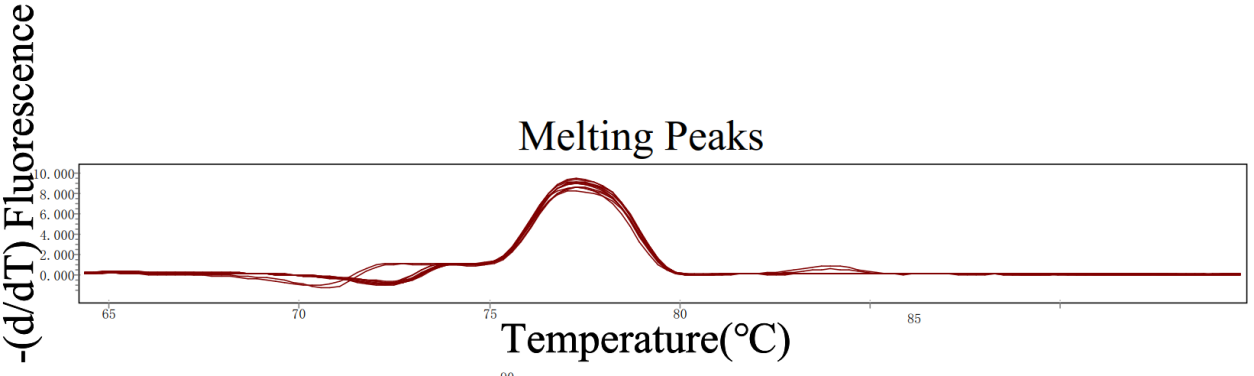


**C**


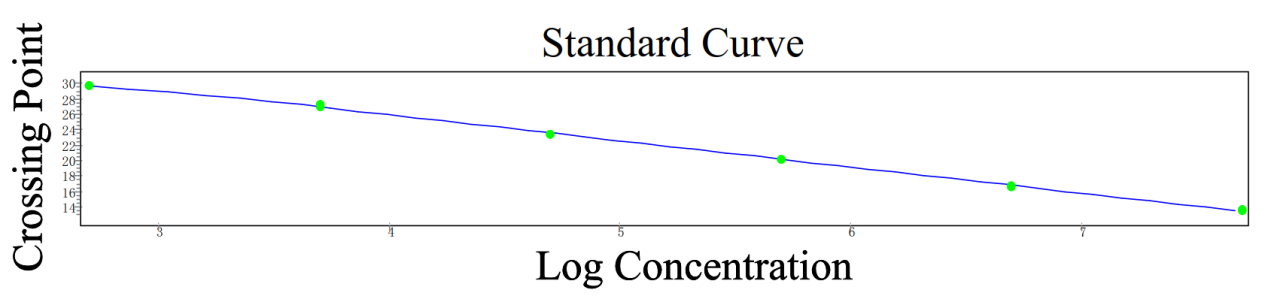
**Fig S1. Quantitative Methylation Specific PCR (QMSP) for the detection of *PDCD-1* methylation in standard samples.** (A) Amplification curves of *PDCD-1* gene methylation standards. (B) Melting curves of serial dilutions of methylated DNA from the *PDCD-1* gene. (C) Standard curve used in the QMSP assay of *PDCD-1* gene.


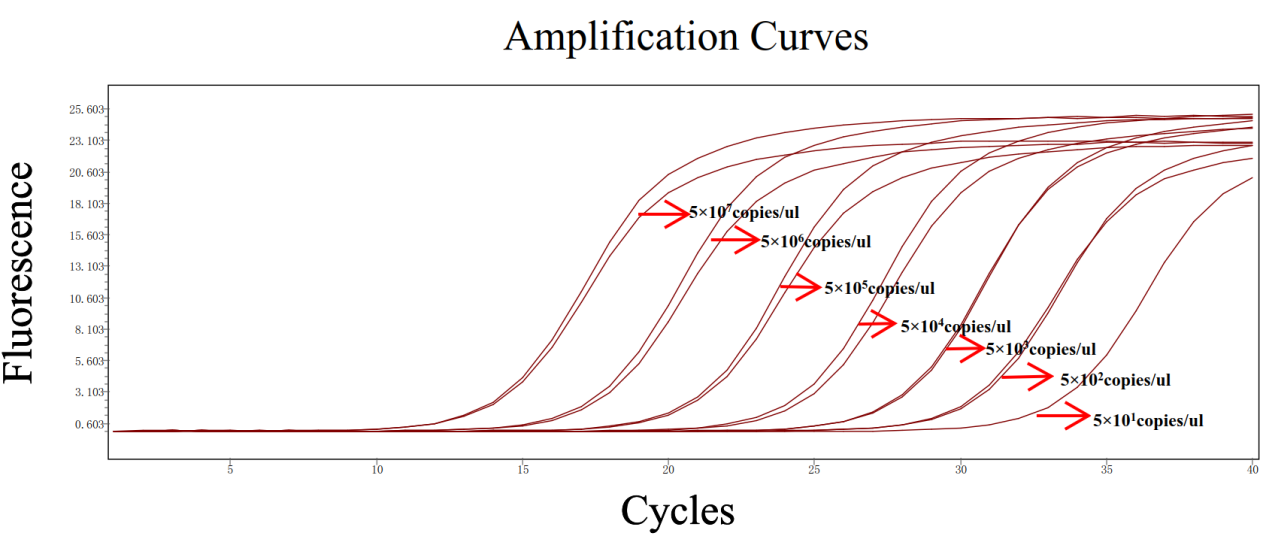

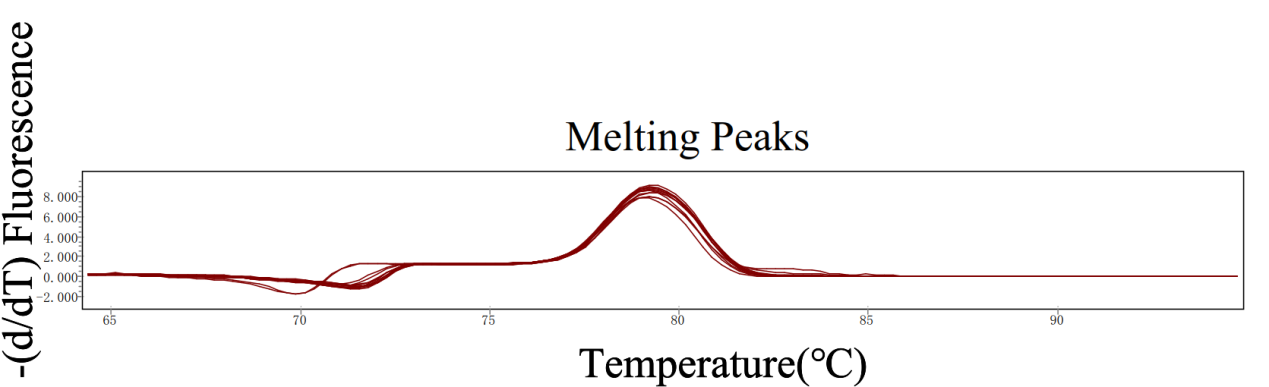


**A**

**C**

**B**


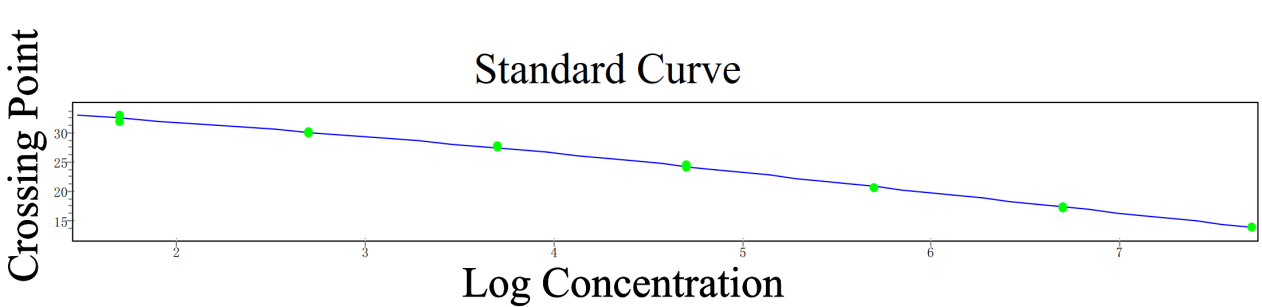


**Fig S2. Quantitative Methylation Specific PCR (QMSP) for the detection of *LAG-3* methylation in standard samples.** (A) Amplification curves of *LAG-3* gene methylation standards. (B) Melting curves of serial dilutions of methylated DNA from the *LAG-3* gene. (C) Standard curve used in the QMSP assay of *LAG-3* gene.


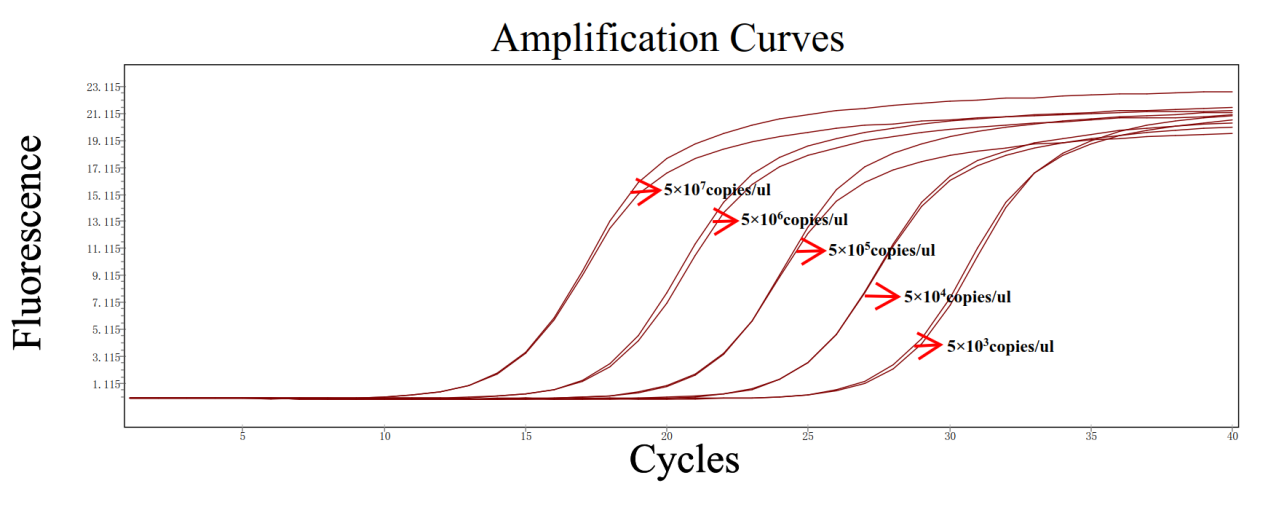


**A**

**B**


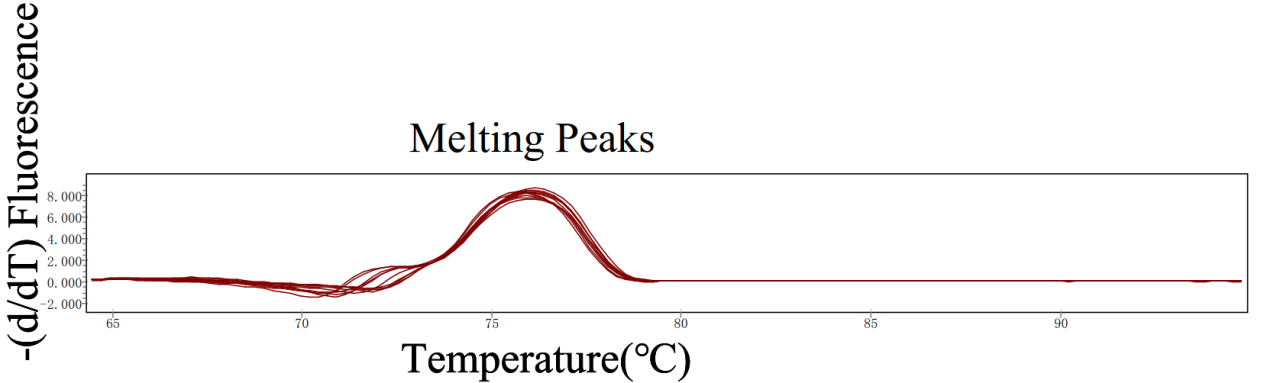


**C**


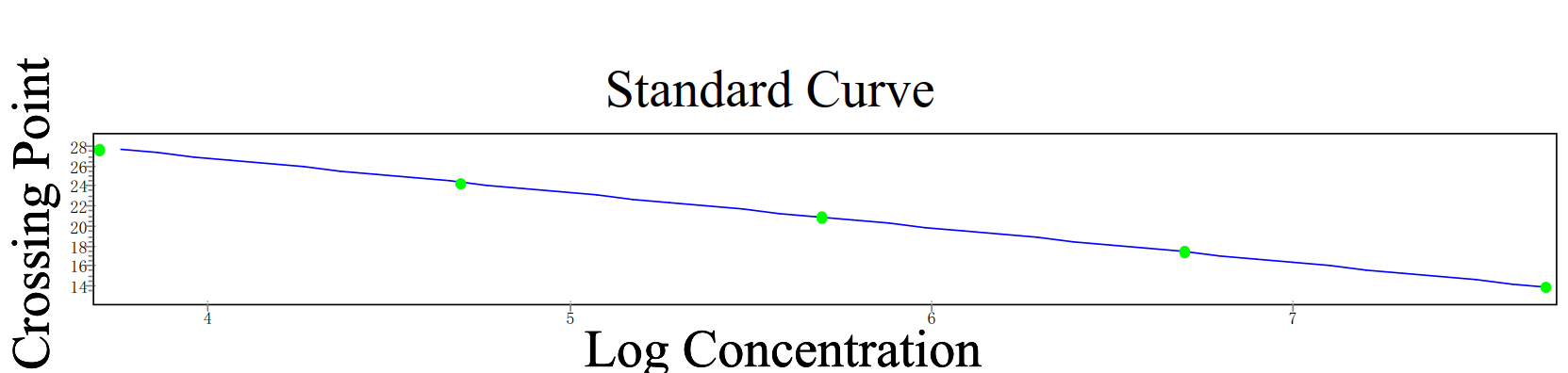


**Fig S3. Quantitative Methylation Specific PCR (QMSP) for the detection of *ACTB* methylation in standard samples.** (A) Amplification curves of *ACTB* gene methylation standards. (B) Melting curves of serial dilutions of methylated DNA from the *ACTB* gene. (C) Standard curve used in the QMSP assay of *ACTB* gene.

###
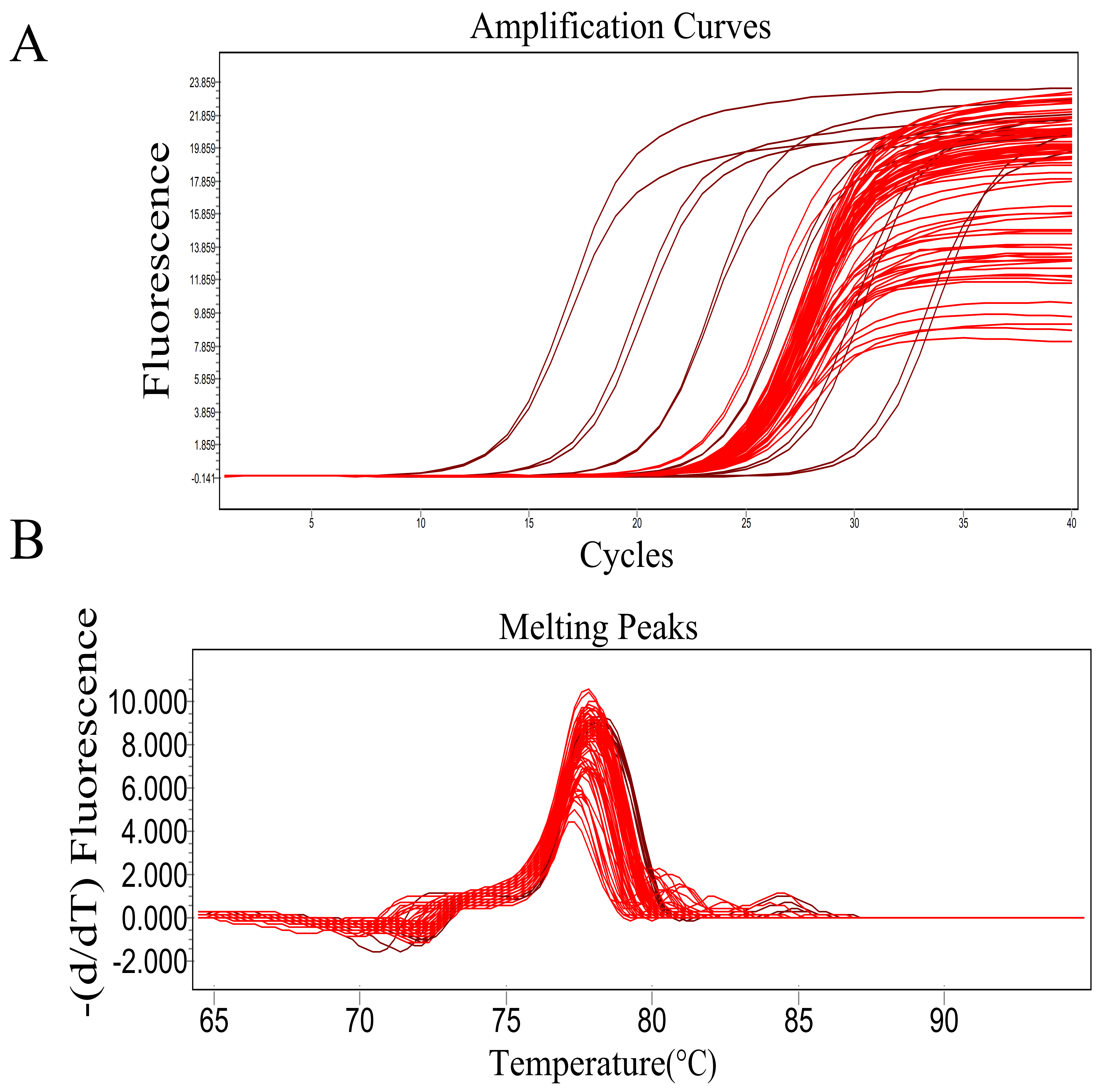
Fig S4. Quantitative Methylation Specific PCR (QMSP) for the detection of *PDCD-1* methylation in PBL samples. (A) Amplification curves used in the *PDCD-1* QMSP assay. (B) Melting curves of PBL samples from the *PDCD-1* gene.

###
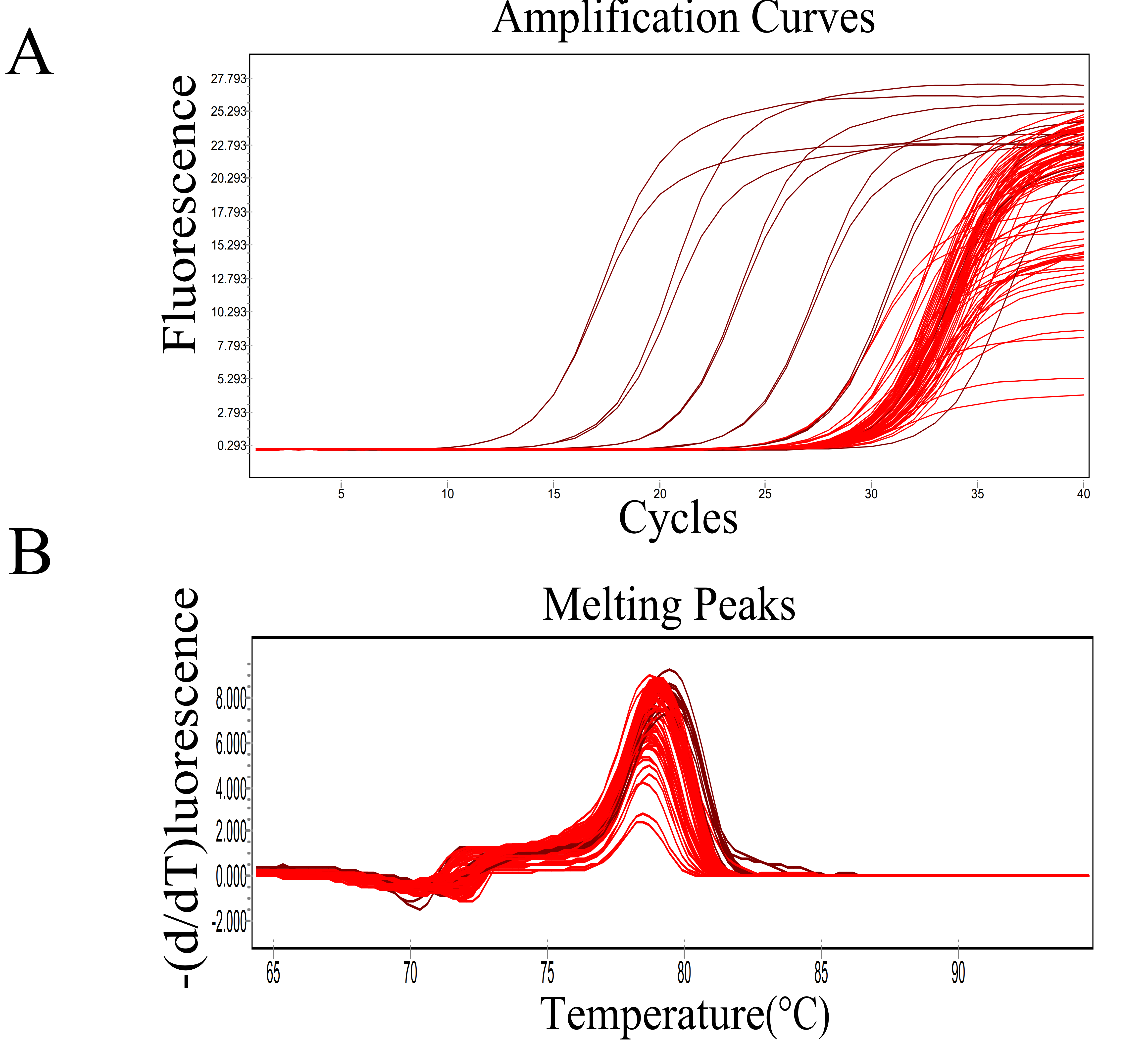


### Fig S5. Quantitative Methylation Specific PCR (QMSP) for the detection of *LAG-3* methylation in PBL samples. (A) Amplification curves used in the *LAG-3* QMSP assay. (B) Melting curves of PBL samples from the *LAG-3* gene.


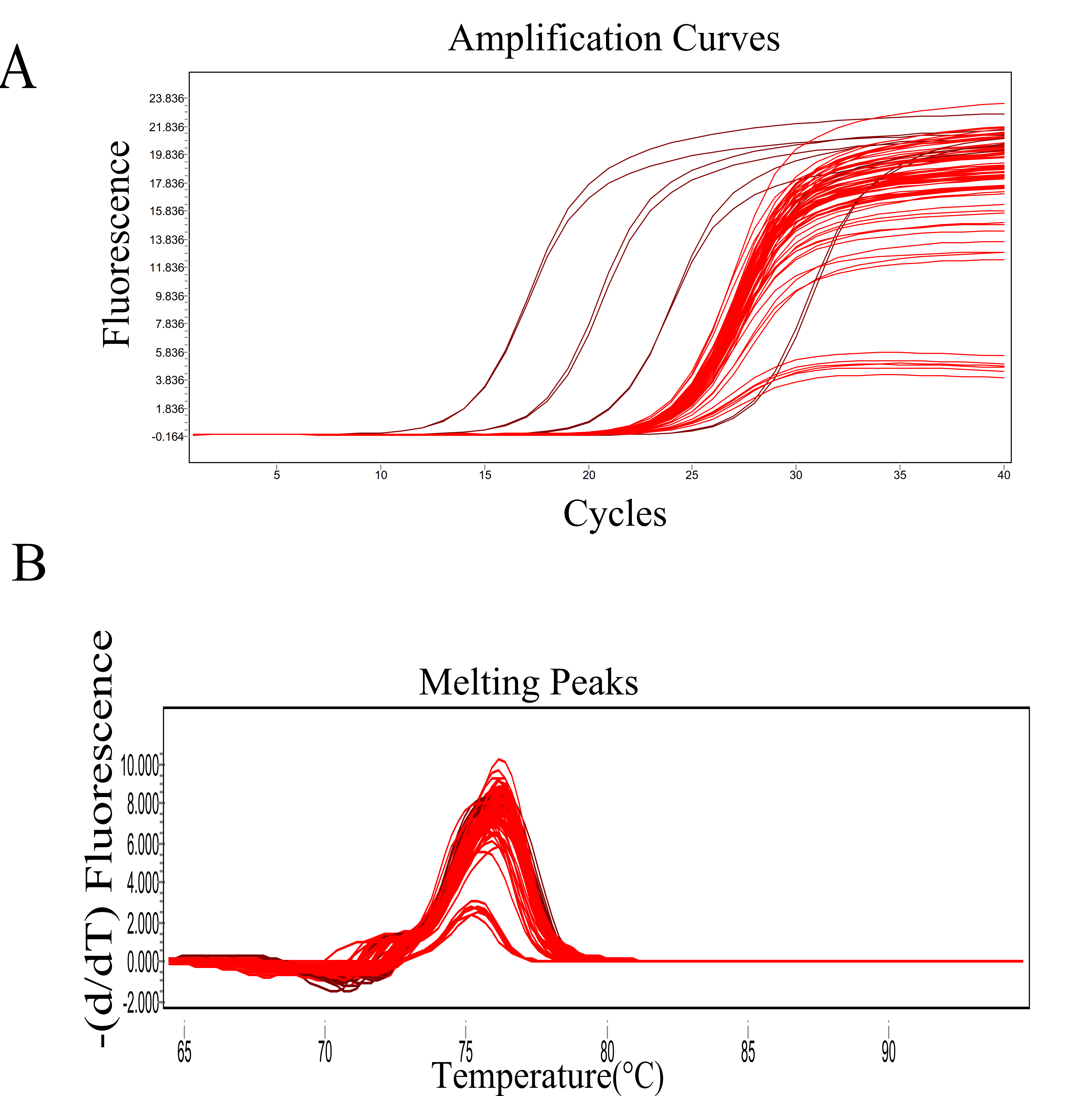


### Fig S6. Quantitative Methylation Specific PCR (QMSP) for the detection of *ACTB* methylation in PBL samples. (A) Amplification curves used in the *ACTB* QMSP assay. (B) Melting curves of PBL samples from the *ACTB* gene.


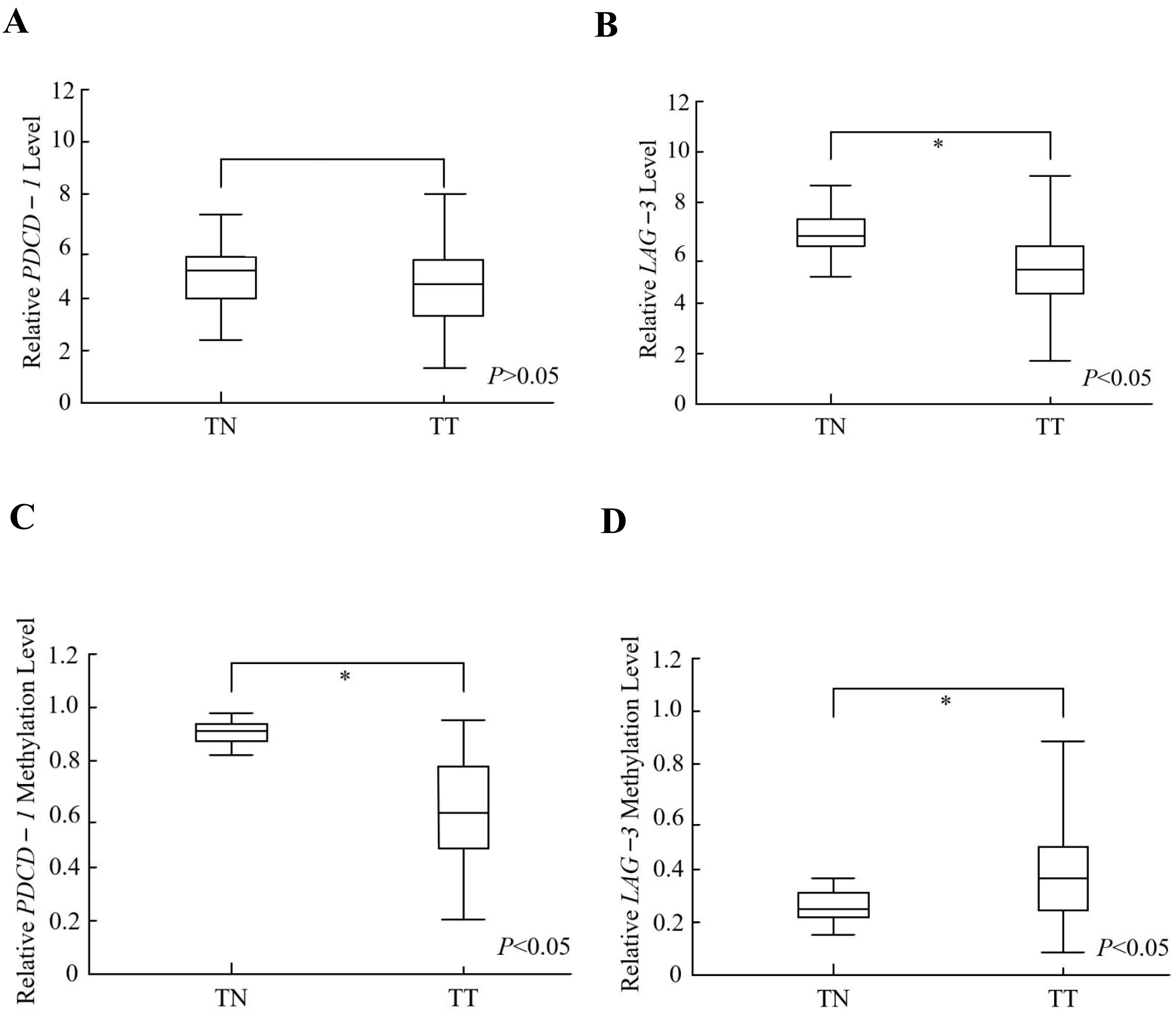


### Fig S7. Gene expression and DNA methylation levels of *PDCD-1*, *LAG-3* in TCGA database. (A) Expression of *PDCD-1* gene. (B) Expression of *LAG-3* gene. (C) Methylation level of *PDCD-1* gene. (D) Methylation level of *LAG-3* gene.


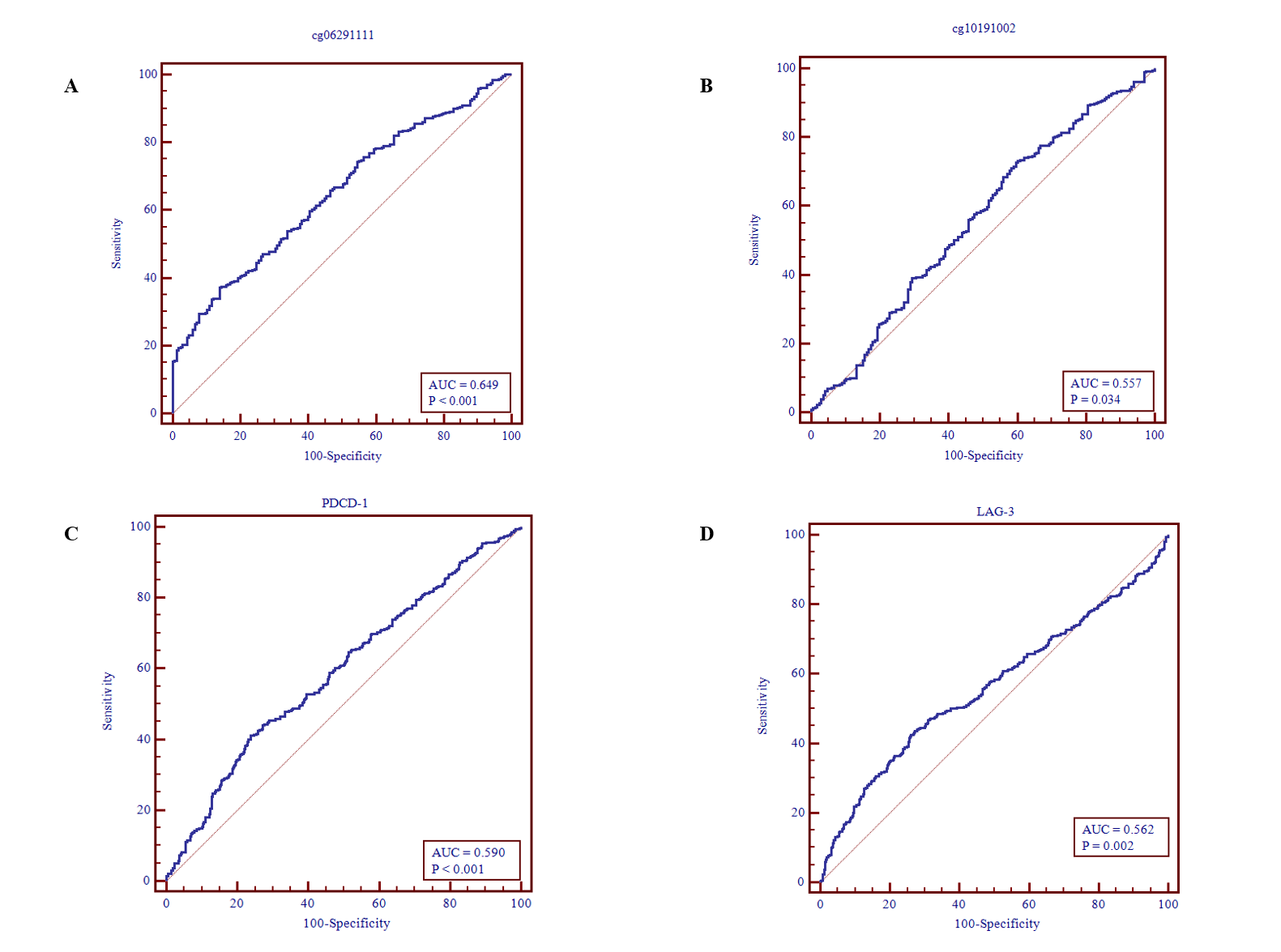


### Fig S8. ROC curves of *PDCD-1*, *LAG-*3 in the case-control study. (A) cg06291111. (B) cg10191002 (C) *PDCD-1* gene (D) *LAG-3* gene

**Table S1.** Primer sequence, amplicon size and reaction conditions.

| **Gene** | **Forward primer** | **Reverse primer** | **Amplicon size** | **Thermal cycling condition** |
| --- | --- | --- | --- | --- |
| *PDCD-1* | 5'-CGGATAAGTTGGTCGTTTT-3' | 5’-AATACCGCTATCATTACGC-3' | 134bp | 95℃ 10min; 95℃ 10s, 58℃ 30s, 72℃ 15s, 40 cycles; 72℃ 5min |
| *LAG-3* | 5'-CGTAAGTTTGGGGTTTGATCG-3' | 5'-CGAACGACACACCGTATTC-3' | 95bp | 95℃ 10min; 95℃ 10s, 62℃ 30s, 72℃ 15s, 40 cycles; 72℃ 5min |
| *ACTB* | 5’-TAGGTATTTTAGTTTTTTTGGGTTT-3’ | 5’-ACCCCAATAAAACATAACACC-3’ | 100bp | 95℃ 10min; 95℃ 10s, 60℃ 30s, 72℃ 15s, 40 cycles; 72℃ 5min |

**Table S2**. Distribution of the basic characteristics of CRC patients and controls in in GEO dataset.

| **Variables** | **CRCs (n=166), n%** | **Controls (n=424), n%** | ****P* value** |
| --- | --- | --- | --- |
| **Age(years)** |  |  | **0.069** |
| **Mean ± SD** | 55.08 ± 6.71 | 53.22 ± 7.19 |  |
| ≤50 | 38 (22.89%) | 144 (33.96%) |  |
| 50- | 95 (57.23%) | 214 (50.47%) |  |
| 60- | 32 (19.28%) | 64 (15.10%) |  |
| >70 | 1 (0.60%) | 2 (0.47%) |  |
| **Gender** |  |  | **0.000** |
| Male | 87 (52.41%) | 84 (19.81%) |  |
| Female | 79 (47.59%) | 340 (80.19%) |  |

CRC: Colorectal Cancer; OR_adj_: Adjusted for age. OR, odds ratio; CI, confidence interval. **P* values < 0.05 were considered statistically significant.

| **Table S3.** Distribution of the environmental factors of the CRC patients and controls. | | | | | | |
| --- | --- | --- | --- | --- | --- | --- |
| **Environmental factors** | **CRCs (n=361), n%** | **Conrtols (n=358), n%** | **OR (95% CI)** | *** *P* value** | **OR_adj_ (95% CI)** | *** *P* value** |
| **Smoking** |  |  |  |  |  |  |
| No | 253 (70.08%) | 269 (75.14%) |  |  |  |  |
| Yes | 108 (29.92%) | 89 (24.86%) | 1.284 (0.777-2.120) | 0.307 | 1.371 (0.821-2.291) | 0.211 |
| **Alcohol drinking** |  |  |  |  |  |  |
| No | 204 (56.51%) | 252 (70.39%) |  |  |  |  |
| Yes | 157 (43.49%) | 106 (29.61%) | 1.825 (1.325-2.512) | **<0.0001** | 1.798 (1.300-2.487) | **<0.0001** |
| **Family history of CRC** |  |  |  |  |  |  |
| No | 318 (88.09%) | 328 (91.62%) |  |  |  |  |
| Yes | 43 (11.91%) | 30 (8.38%) | 1.486 (0.909-2.429) | 0.114 | 1.639 (0.992-2.7097) | 0.054 |
| **Overnight food** |  |  |  |  |  |  |
| No | 128 (35.46%) | 167 (46.65%) |  |  |  |  |
| Yes | 233 (64.54%) | 191 (53.35%) | 1.593 (1.153-2.200) | **0.005** | 1.477 (1.061-2.05) | **0.021** |
| **Salty taste** |  |  |  |  |  |  |
| Slight | 176 (48.75%) | 247 (68.99%) |  |  |  |  |
| Heavy | 185 (51.25%) | 111 (31.01%) | 2.130 (1.524-2.977) | **<0.0001** | 2.143 (1.518-3.024) | **<0.0001** |
| **Hot-scalded food** |  |  |  |  |  |  |
| No | 252 (69.81%) | 299 (83.52%) |  |  |  |  |
| Yes | 109 (30.19%) | 59 (16.48%) | 2.214 (1.531-3.201) | **<0.0001** | 2.170 (1.488-3.164) | **<0.0001** |
| **Coarse grain** |  |  |  |  |  |  |
| No | 110 (30.47%) | 97 (27.09%) |  |  |  |  |
| Yes | 251 (69.53%) | 261 (72.91%) | 0.844 (0.596-1.196) | 0.339 | 0.870 (0.608-1.246) | 0.445 |
| **Egg, numbers/week** |  |  |  |  |  |  |
| <3 | 276 (76.45%) | 157 (43.85%) |  |  |  |  |
| ≥3 | 85 (23.55%) | 201(56.16%) | 0.242 (0.156-0.375) | **<0.0001** | 0.230 (0.146-0.364) | **<0.0001** |
| **Pork, times/week** |  |  |  |  |  |  |
| <3 | 130 (36.01%) | 188 (52.51%) |  |  |  |  |
| ≥3 | 231 (63.99%) | 170 (47.49%) | 1.966 (1.404-2.754) | **<0.0001** | 1.924 (1.375-2.691) | **<0.0001** |
| **Beef, times/week** |  |  |  |  |  |  |
| <3 | 145 (40.17%) | 248 (69.27%) |  |  |  |  |
| ≥3 | 216 (59.83%) | 110 (30.73%) | 3.342 (2.422-4.612) | **<0.0001** | 3.384 (2.436-4.701) | **<0.0001** |
| **Fowl, times/week** |  |  |  |  |  |  |
| <3 | 221 (61.22%) | 283 (79.05%) |  |  |  |  |
| ≥3 | 140 (38.78%) | 75 (20/95%) | 2.417 (1.645-3.551) | **<0.0001** | 2.480 (1.679-3.665) | **<0.0001** |
| **Milk** |  |  |  |  |  |  |
| No | 201 (55.68%) | 205 (57.26%) |  |  |  |  |
| Yes | 160 (44.32%) | 153 (42.74%) | 1.071 (0.738-1.555) | 0.710 | 1.065 (0.724-1.566) | 0.744 |
| **Garlic** |  |  |  |  |  |  |
| No | 114 (31.58%) | 119 (33.24%) |  |  |  |  |
| Yes | 247 (68.42%) | 239 (66.76%) | 1.081 (0.765-1.526) | 0.657 | 1.152 (0.811-1.635) | 0.427 |
| **Pickled cabbage** |  |  |  |  |  |  |
| No | 121(33.52%) | 170 (47.49%) |  |  |  |  |
| Yes | 240(66.48%) | 188 (52.51%) | 1.790 (1.299-2.467) | **<0.0001** | 1.718 (1.219-2.420) | **0.002** |
| **Smoked and baked food** |  |  |  |  |  |  |
| No | 262 (72.58%) | 286 (79.89%) |  |  |  |  |
| Yes | 99 (27.42%) | 72 (20.11%) | 1.502 (1.006-2.244) | **0.047** | 1.793 (1.201-2.677) | **0.005** |
| **Deep-sea fish** |  |  |  |  |  |  |
| No | 136 (37.67%) | 129 (36.03%) |  |  |  |  |
| Yes | 225 (62.33%) | 229 (63.97%) | 0.934 (0.649-1.344) | 0.708 | 0.946 (0.658-1.360) | 0.761 |
| **Fried food** |  |  |  |  |  |  |
| No | 291 (80.61%) | 283 (79.05%) |  |  |  |  |
| Yes | 70 (19.39%) | 75 (20.95%) | 0.915 (0.564-1.485) | 0.711 | 0.924 (0.617-1.552) | 0.924 |

CRC: Colorectal Cancer; OR_adj_: Adjusted for age. OR, odds ratio; CI, confidence interval. **P* values < 0.05 were considered statistically significant.

| **Table S4.** Association between CRC risk and *PDCD-1*, *LAG-3* methylation levels in PBL calculated by using the average of coefficients and slopes of all standard curves. | | | | | | |
| --- | --- | --- | --- | --- | --- | --- |
| **Gene** | **Hypomethylation** | **Hypermethylation** | **OR (95% CI)** | *** *P* value** | **OR_adj_ (95% CI)** | *** *P* value** |
| ***PDCD-1*** |  |  |  |  |  |  |
| CRCs (n=389), n% | 217 (55.78%) | 172 (44.22%) |  | **<0.0001** |  | **<0.0001** |
| Controls (n=386), n% | 129 (33.42%) | 257 (66.58%) | 0.398 (0.297-0.532) |  | 0.392 (0.287-0.534) |  |
| ***LAG-3*** |  |  |  |  |  |  |
| CRCs (n=390), n% | 290 (53.59%) | 181 (46.41%） |  | **<0.0001** |  | **<0.0001** |
| Controls (n=397), n% | 140 (35.26%) | 257 (64.74%) | 0.472 (0.354-0.628) |  | 0.452 (0.332-0.614) |  |

OR_adj_: Adjusted for age, gender. CRC: Colorectal Cancer; OR, odds ratio; CI, confidence interval. **P* values < 0.5 were considered statistically significant.

**Table S5.** Association between the methylation levels of *PDCD-1*, *LAG-3* and CRC risk stratified by age in GEO dataset.

| **Gene** | **No. of Hypermethylation (CRCs/Controls)** | **No. of Hypomethylation**  **(CRCs/Controls)** | **OR (95% CI)** | ***P* value**^*^ | ***P* value**^†^ |
| --- | --- | --- | --- | --- | --- |
| ***PDCD-1*** |  |  |  |  |  |
| Age, years |  |  |  |  |  |
| <60 | 18/129 | 110/219 | 0.278 (0.161-0.478) | **<0.0001** |  |
| ≥60 | 6/28 | 32/48 | 0.321 (0.120-0.864) | **0.024** | 0.880 |
| ***LAG-3*** |  |  |  |  |  |
| Age, years |  |  |  |  |  |
| <60 | 82/256 | 46/92 | 0.641 (0.416-0.987) | 0.044 |  |
| ≥60 | 18/52 | 20/24 | 0.415 (0.187-0.924) | 0.031 | 0.350 |

CRC, Colorectal Cancer; OR, odds ratio; CI, confidence interval.

^*^*P* values were calculated using Logistic regression analysis, *P* values < 0.025 were considered statistically significant.

†Test for heterogeity between ORs was conducted by fixed effect models with STATA (version 14), *P* values < 0.05 were considered statistically significant.

**Table S6.** Association between the methylation levels of *PDCD-1*, *LAG-3* and CRC risk stratified by different environmental factors in case-control study.

|  | ***LAG-3*** | | | | ***PDCD-1*** | | | |
| --- | --- | --- | --- | --- | --- | --- | --- | --- |
| **Environmental factors** | **No. of hype (CRCs /Controls)** | **No. of hypo (CRCs /Controls)** | **OR_adj_ (95% CI)** | *** *P* value** | **No. of hype (CRCs /Controls)** | **No. of hypo (CRCs /Controls)** | **OR_adj_ (95% CI)** | *** *P* value** |
| Alcohol drinking |  |  |  |  |  |  |  |  |
| No | 118/192 | 86/60 | 0.401 (0.337-0.476) | <0.0001 | 117/190 | 87/62 | 0.434 (0.366-0.514) | <0.0001 |
| Yes | 86/29 | 71/77 | 0.433 (0.346-0.543) | <0.0001 | 95/81 | 62/25 | 0.426 (0.336-0.541) | <0.0001 |
| Salty taste |  |  |  |  |  |  |  |  |
| Slight | 112/186 | 73/61 | 0.500 (0.420-0.594) | <0.0001 | 102/192 | 83/55 | 0.347 (0.291-0.414) | <0.0001 |
| Heavy | 92/28 | 84/83 | 0.339 (0.272-0.424) | <0.0001 | 110/79 | 66/32 | 0.683 (0.551-0.848) | 0.001 |
| Hot-scalded food |  |  |  |  |  |  |  |  |
| No | 139/225 | 113/74 | 0.407 (0.351-0.474) | <0.0001 | 147/225 | 105/74 | 0.461 (0.397-0.573) | <0.0001 |
| Yes | 65/44 | 44/15 | 0.334 (0.241-0.463) | <0.0001 | 65/46 | 45/13 | 0.404 (0.293-0.557) | <0.0001 |
| Egg, numbers/week |  |  |  |  |  |  |  |  |
| <3 | 56/144 | 29/57 | 0.702 (0.555-0.888) | 0.003 | 51/154 | 34/47 | 0.471 (0.373-0.595) | <0.0001 |
| ≥3 | 148/125 | 128/32 | 0.291 (0.240-0.354) | <0.0001 | 161/117 | 115/40 | 0.453 (0.376-0.546) | <0.0001 |
| Pork, times/week |  |  |  |  |  |  |  |  |
| <3 | 81/150 | 49/38 | 0.418 (0.339-0.516) | <0.0001 | 79/139 | 51/49 | 0.540 (0.441-0.660) | <0.0001 |
| ≥3 | 123/119 | 128/51 | 0.454 (0.380-0.542) | <0.0001 | 133/132 | 98/38 | 0.393 (0.326-0.474) | <0.0001 |
| Beef, times/week |  |  |  |  |  |  |  |  |
| <3 | 94/187 | 51/61 | 0.607 (0.503-0.733) | <0.0001 | 84/193 | 61/55 | 0.396 (0.328-0.478) | <0.0001 |
| ≥3 | 110/82 | 106/28 | 0.327 (0.264-0.406) | <0.0001 | 128/78 | 88/32 | 0.592 (0.481-0.729) | <0.0001 |
| Fowl, times/week |  |  |  |  |  |  |  |  |
| <3 | 125/210 | 95/73 | 0.438 (0.373-0.515) | <0.0001 | 122/210 | 98/73 | 0.425 (0.362-0.500) | <0.0001 |
| ≥3 | 79/59 | 62/16 | 0.319 (0.244-0.418) | <0.0001 | 90/61 | 51/14 | 0.411 (0.311-0.544) | <0.0001 |
| Pickled cabbage |  |  |  |  |  |  |  |  |
| No | 69/120 | 52/50 | 0.543 (0.422-0.667) | <0.0001 | 67/132 | 54/38 | 0.347 (0.279-0.430) | <0.0001 |
| Yes | 135/149 | 105/39 | 0.315 (0.261-0.380) | <0.0001 | 145/139 | 95/49 | 0.531 (0.445-0.635) | <0.0001 |
| Smoked and baked food |  |  |  |  |  |  |  |  |
| No | 155/215 | 107/71 | 0.488 (0.418-0.570) | <0.0001 | 161/221 | 101/65 | 0.457 (0.390-0.536) | <0.0001 |
| Yes | 49/54 | 50/18 | 0.237 (0.175-0.321) | <0.0001 | 51/50 | 48/22 | 0.497 (0.378-0.655) | <0.0001 |

CRC, Colorectal Cancer; OR, odds ratio; CI, confidence interval.

^*^*P* values were calculated using Logistic regression analysis, *P* values < 0.025 were considered statistically significant.

| **Table S7.** Effects of combination and interaction between environmental factors and methylation of *PDCD-1* on the risk of CRC. | | | | |
| --- | --- | --- | --- | --- |
| **Environmental factors** | **Hypermethylation** | **Hypomethylation** | **Interaction** | |
|  | **OR_eg_ (95% CI)** | | **OR_i_ (95% CI)** | *** *P* value** |
| **Alcohol drinking** |  |  |  |  |
| No |  | 2.268 (1.509-3.409) |  |  |
| Yes | 1.892 (1.270-2.820) | **4.032 (2.380-6.832)** | 0.939 (0.455-1.938) | 0.865 |
| **Overnight food** |  |  |  |  |
| No |  | 1.453 (0.836-2.526) |  |  |
| Yes | 1.273 (0.875-1.851) | **3.743 (2.332-6.007)** | 2.024 (0.943-4.346) | 0.070 |
| **Salty taste** |  |  |  |  |
| Slight |  | 2.779 (1.739-4.441) |  |  |
| Heavy | 2.606 (1.774-3.827) | **3.903 (2.229-6.836)** | 0.539 (0.220-1.321) | 0.168 |
| **Hot-scalded food** |  |  |  |  |
| No |  | 2.143 (1.438-3.193) |  |  |
| Yes | 2.162 (1.379-3.391) | **5.268 (2.650-10.471)** | 1.137 (0.440-2.937) | 0.788 |
| **Egg, numbers/week** |  |  |  |  |
| <3 |  | 2.099 (1.312-3.358) |  |  |
| ≥3 | 0.245 (0.137-0.440) | 0.529 (0.240-1.166) | 1.029 (0.304-3.480) | 0.961 |
| **Pork, times/week** |  |  |  |  |
| <3 |  | 1.871 (1.123-3.118) |  |  |
| ≥3 | 1.798 (1.228-2.632) | **4.505 (2.777-7.309)** | 1.339 (0.659-2.724) | 0.418 |
| **Beef, times/week** |  |  |  |  |
| <3 |  | 2.524 (1.542-4.131) |  |  |
| ≥3 | 3.730 (2.457-5.663) | **6.271 (3.872-10.156)** | 0.666 (0.306-1.451) | 0.301 |
| **Fowl, times/week** |  |  |  |  |
| <3 |  | 2.326 (1.577-3.429) |  |  |
| ≥3 | 2.559 (1.696-3.862) | **6.215 (3.118-12.389)** | 1.044 (0.455-2.397) | 0.918 |
| **Pickled cabbage** |  |  |  |  |
| No |  | 2.742 (1.584-4.744) |  |  |
| Yes | 2.028 (1.351-3.045) | **3.822 (2.389-6.115)** | 0.687 (0.327-1.445) | 0.319 |
| **Smoked and baked food** |  |  |  |  |
| No |  | 2.145 (1.435-3.206) |  |  |
| Yes | 1.425 (0.893-2.275) | **2.924 (****1.493-5.725）** | 0.956 (0.358-2.557) | 0.927 |

CRC: Colorectal Cancer; OR, odds ratio; CI, confidence interval. **P* values < 0.05 were considered statistically significant.

| **Table S8.** Effects of combination and interaction between environmental factors and methylation of *LAG-3* on the risk of CRC. | | | | |
| --- | --- | --- | --- | --- |
| **Environmental factors** | **Hypermethylation** | **Hypomethylation** | **Interaction** | |
|  | **OR_eg_ (95% CI)** | | **OR_i_ (95% CI)** | *** *P* value** |
| **Alcohol drinking** |  |  |  |  |
| No |  | 2.321 (1.547-3.482) |  |  |
| Yes | 1.806 (1.203-2.712) | **4.008 (2.453-6.548)** | 0.956 (0.476-1.919) | 0.899 |
| **Overnight food** |  |  |  |  |
| No |  | 1.663 (1.023-2.706) |  |  |
| Yes | 1.337 (0.888-2.011) | **4.292 (2.646-6.964)** | 1.931 (0.989-3.768) | 0.054 |
| **Salty taste** |  |  |  |  |
| Slight |  | 1.983 (1.312-2.998) |  |  |
| Heavy | 1.848 (1.225-2.786) | **5.031 (3.046-8.308)** | 1.373 (0.705-2.677) | 0.352 |
| **Hot-scalded food** |  |  |  |  |
| No |  | 2.465 (1.713-3.549) |  |  |
| Yes | 2.400 (1.523-3.783) | **4.908 (2.603-9.430)** | 0.830 (0.370-1.859) | 0.650 |
| **Egg, numbers/week** |  |  |  |  |
| <3 |  | 3.347 (2.125-5.271) |  |  |
| ≥3 | 0.337 (0.205-40.554) | **0.439 (0.233-0.827)** | **0.389 (0.190-0.794)** | **0.010** |
| **Pork, times/week** |  |  |  |  |
| <3 |  | 2.370 (1.412-3.978) |  |  |
| ≥3 | 1.907 (1.285-2.830) | **3.920 (2.503-6.139)** | 0.867 (0.438-1.717) | 0.683 |
| **Beef, times/week** |  |  |  |  |
| <3 |  | 1.652 (1.027-2.655) |  |  |
| ≥3 | 2.642 (1.740-4.013) | **7.563 (4.616-12.392)** | 1.733 (0.825-3.643) | 0.146 |
| **Fowl, times/week** |  |  |  |  |
| <3 |  | 2.187 (1.494-3.202) |  |  |
| ≥3 | 2.247 (1.412-3.578) | **6.508 (3.505-12.084)** | 1.324 (0.619-2.834) | 0.469 |
| **Pickled cabbage** |  |  |  |  |
| No |  | 1.806 (1.080-3.019) |  |  |
| Yes | 1.571 (1.061-2.327) | **4.663 (2.836-7.667)** | 1.644 (0.784-3.444) | 0.186 |
| **Smoked and baked food** |  |  |  |  |
| No |  | 2.071 (1.419-3.023) |  |  |
| Yes | 1.238 (0.706-2.170) | **3.980 (2.114-7.495)** | 1.552 (0.559-4.019) | 0.356 |

CRC: Colorectal Cancer; OR, odds ratio; CI, confidence interval. **P* values < 0.05 were considered statistically significant.
